# Infestation patterns and ecological distribution of fleas and sucking lice on *Rattus tanezumi* in southwest China: Evidence from a long-term multi-provincial study (2000–2024)

**DOI:** 10.14202/vetworld.2026.191-209

**Published:** 2026-01-20

**Authors:** Xue-Jiao Zhu, Ya-Nan Li, Xian-Guo Guo, Tian-Guang Ren, Yong-Guang Jing, Lei Zhang, Ti-Jun Qian

**Affiliations:** 1 Institute of Pathogens and Vectors, Key Laboratory of Ectoparasite Systematics and Evolution of Yunnan Provincial Education Department, Yunnan Provincial Key Laboratory for Zoonosis Control and Prevention, Dali University, Dali, Yunnan, 671000, China; 2 School of Government Administration, Baoshan University, Baoshan, Yunnan, 678000, China

**Keywords:** ecological distribution, ectoparasitic insects, fleas, *Rattus tanezumi*, rodent hosts, southwest China, sucking lice, zoonotic vectors

## Abstract

**Background and Aim:**

The oriental house rat (*Rattus tanezumi*) is a dominant commensal rodent in southwest China and an important reservoir host for multiple zoonotic pathogens. Fleas and sucking lice that parasitize this species play a critical role in the maintenance and transmission of flea-borne and louse-associated diseases. However, long-term, large-scale evidence on the infestation patterns, ecological distribution, and host–parasite relationships of these ectoparasites remains limited. This study aimed to comprehensively characterize the infestation status, community structure, and ecological determinants of fleas and sucking lice on *R. tanezumi* across southwest China.

**Materials and Methods:**

A retrospective analysis was conducted using data from systematic field investigations conducted at 116 survey sites across five provincial regions of southwest China between 2000 and 2024. Rodents were captured using standardized trapping protocols in indoor and outdoor habitats. Fleas and sucking lice were collected, mounted, and taxonomically identified under a microscope. Infestation indices, including prevalence, mean abundance, and mean intensity, were calculated. Community diversity indices, host-related factors (sex, age, and relative fatness), environmental gradients (latitude, longitude, and altitude), and habitat types were analyzed. Association coefficients and Spearman’s rank correlation were used to assess interspecific and intergroup relationships.

**Results:**

A total of 3,069 *R. tanezumi* were examined, of which 40.40% were infested with ectoparasitic insects. Overall, 12,539 insects belonging to 34 species were identified, comprising 30 flea species and four sucking louse species. Fleas exhibited markedly higher species diversity but lower individual abundance than sucking lice. Ten flea species are known or potential vectors of zoonotic pathogens. Sucking lice showed significantly higher infestation prevalence and intensity than fleas (p < 0.05). Male, adult, and low-fatness hosts harbored significantly heavier louse infestations, whereas flea infestation showed no clear sex or age bias. Infestation indices varied significantly across environmental gradients and habitats. The association coefficient between fleas and lice was close to zero, indicating mutual independence.

**Conclusion:**

*R. tanezumi* harbors a diverse assemblage of ectoparasitic insects, including multiple zoonotic flea species. Fleas and sucking lice exhibit contrasting community structures, host associations, and ecological patterns. These findings provide long-term, multi-regional evidence supporting targeted surveillance and control strategies for rodent-associated ectoparasites and related zoonoses in southwest China.

## INTRODUCTION

The oriental house rat (Asian house rat), *Rattus tanezumi* (Temminck 1844), is a widespread rodent species distributed throughout many regions of China, particularly in southern and southwestern areas, and belongs to the family Muridae within the order Rodentia [1–3]. *Rattus flavipectus* (Milne-Edwards, 1871) is a commonly used synonym for *R. tanezumi* and continues to appear in several published studies [4–6]. Rodents commonly harbor two major groups of ectoparasitic insects on their body surface: fleas (Order Siphonaptera) and sucking lice (Order Phthiraptera). As prevalent ectoparasites, fleas and lice can cause irritation in animals and humans through biting, and some flea species may directly parasitize humans, resulting in tungiasis. More importantly, fleas act as vectors of several zoonotic diseases, including plague, murine typhus, flea-borne spotted fever, and bartonellosis. The causative agents of these diseases (*Yersinia pestis*, *Rickettsia mooseri*, *Rickettsia felis*, and *Bartonella* spp.) can be transmitted among rodent hosts and from rodents to humans and domestic animals through the blood-feeding activity of vector fleas [7–16]. Certain flea species also serve as intermediate hosts of tapeworms such as *Hymenolepis nana*, *Hymenolepis diminuta*, and *Dipylidium caninum*, which may cause taeniasis in pets, livestock, and humans [10, 11, 14]. In addition, fleas have been implicated in the transmission of tularemia, leptospirosis, rabbit myxomatosis, trench fever, feline leukemia, mycoplasmal infections, and Lyme disease [10, 13, 15]. The human louse, *Pediculus humanus*, is a well-recognized vector of epidemic typhus, epidemic relapsing fever, and trench fever [12, 17–19]. Although rodent lice do not directly transmit pathogens to humans, they can function as reservoir hosts for several zoonotic agents, including *Yersinia pestis*, *Francisella tularensis*, and *Rickettsia mooseri* [19–22].

Southwest China, encompassing Yunnan, Guizhou, Sichuan, Chongqing, and Xizang, represents a natural focus for numerous zoonotic diseases, such as plague, murine typhus, bartonellosis, leptospirosis, hemorrhagic fever with renal syndrome (HFRS), and scrub typhus [23–31]. *R. tanezumi* is one of the dominant rodent species in this region and maintains a close association with human settlements and agricultural environments. As a notorious agricultural pest, this species causes substantial damage to crops and household infrastructure, prompting frequent control campaigns by local authorities [32–34]. In addition, *R. tanezumi* serves as an important reservoir and source of infection for multiple zoonotic diseases, including plague, murine typhus, HFRS, scrub typhus, leptospirosis, bartonellosis, rat-bite fever, and schistosomiasis [2, 3, 32, 33, 35, 36]. Consequently, investigating the infestation and distribution patterns of ectoparasitic insects on *R. tanezumi* in southwest China is of considerable medical and veterinary importance.

Ectoparasites do not constitute a single taxonomic group but include diverse assemblages of small arthropods. Rodent ectoparasites typically include chiggers (trombiculid mites), gamasid mites, sarcoptic mites, demodectic mites, ticks, fleas, and sucking lice [37]. From a taxonomic perspective, mites and ticks belong to the class Arachnida within the phylum Arthropoda, whereas fleas and sucking lice are members of the class Insecta. Each of these arthropod groups contains a large number of species; to date, more than 3,000 chigger species and over 8,000 gamasid mite species have been described [2, 34]. The high species diversity of ectoparasitic arthropods presents substantial challenges for accurate taxonomic identification, often limiting the feasibility of comprehensive investigations encompassing all groups simultaneously. Our research team has previously conducted extensive field surveys in southwest China and reported infestation and distribution patterns of ectoparasitic mites, including chiggers and gamasid mites, on rodents and other sympatric small mammals [38, 39]. In particular, earlier studies documented the infestation ecology of gamasid mites and chiggers on *R. tanezumi* in Yunnan Province and across southwest China during different survey periods [2, 3, 34, 40].

Despite the recognized importance of *R. tanezumi* as a dominant commensal rodent and reservoir host of multiple zoonotic pathogens in southwest China, existing studies have largely focused on host ecology or single groups of ectoparasites. Our previous investigations comprehensively documented the infestation patterns, community structure, and environmental determinants of ectoparasitic mites, particularly chiggers and gamasid mites, on *R. tanezumi* and other sympatric small mammals across southwest China during different survey periods [2, 3, 34, 38–40]. These studies revealed pronounced environmental heterogeneity, host-related biases, and ecological gradients shaping mite infestations. However, ectoparasitic insects, specifically fleas and sucking lice, have not been systematically analyzed alongside mites, largely due to historical limitations in taxonomic identification and manpower. Most earlier reports on fleas in southwest China were geographically restricted, temporally short-term, or focused on single flea species or specific zoonotic agents, while sucking lice were often overlooked or treated only as ancillary findings. Moreover, no long-term, multi-provincial study has simultaneously assessed flea and sucking louse infestations on *R. tanezumi*, nor explored their comparative community structures, host-associated factors (sex, age, and nutritional status), environmental heterogeneity, and potential intergroup relationships. The lack of integrated evidence limits a comprehensive understanding of rodent–ectoparasite systems and constrains accurate risk assessment of flea- and louse-associated zoonoses in this region.

Building on our previous long-term field investigations and ecological analyses of ectoparasitic mites on *R. tanezumi* in southwest China, the present study aimed to address these critical knowledge gaps by conducting a comprehensive retrospective analysis of ectoparasitic insects, namely fleas and sucking lice, on this key rodent host. Specifically, this study aimed to (i) characterize the species composition, infestation indices, and community structure of fleas and sucking lice parasitizing *R. tanezumi* across five provincial regions of southwest China over a 24-year period; (ii) compare infestation patterns between fleas and sucking lice in relation to host biological factors, including sex, age, and relative fatness; (iii) evaluate the influence of environmental gradients and habitat types on insect infestation and distribution; and (iv) analyze mutual relationships and interspecific associations between flea and louse communities on the same host species. By integrating long-term, large-scale ecological data with standardized infestation metrics, this study seeks to provide a more complete understanding of rodent-associated ectoparasitic insects and to generate scientifically robust evidence to support surveillance, prevention, and control strategies for vector-borne zoonotic diseases in southwest China.

## MATERIALS AND METHODS

### Ethical approval

All procedures involving animals in this study were conducted in strict accordance with the Animal Ethics Procedures and Guidelines of the People’s Republic of China and complied with internationally accepted principles for the ethical use of animals in research. The capture, handling, examination, and specimen collection of rodents were formally reviewed and approved by the Animals’ Ethics Committees of Dali Medical College (1990–2000) and Dali University (from 2001 onward), which were responsible for ethical oversight during different phases of this long-term investigation.

Ethical approval was granted under the following approval codes and dates: DLYXY1990-0109 (approved on 9 January 1990), DLXY2001-1116 (approved on 16 November 2001), and DLDXLL2020-1104 (approved on 4 November 2020). These approvals collectively covered all field investigations conducted between 2000 and 2024 across the five provincial regions of southwest China.

Rodent trapping and handling were performed by trained personnel using standardized and humane protocols designed to minimize animal stress and suffering. Captured animals were handled individually, examined promptly, and processed following approved biosafety and animal welfare procedures. All instruments were disinfected between examinations, and appropriate personal protective equipment was used by field and laboratory staff to ensure both animal welfare and investigator safety.

As this study represents a retrospective analysis of data and specimens obtained from long-term routine surveillance and ecological investigations, no experimental manipulation or invasive procedures beyond standard parasitological examination were performed. Representative voucher specimens of rodent hosts and ectoparasitic insects were deposited in the specimen repository of the Institute of Pathogens and Vectors, Dali University, ensuring traceability, reproducibility, and future reference.

### Study design, period, and survey area

This study was a retrospective analysis based on long-term historical field investigations and taxonomic identification conducted at 116 survey sites across five provincial regions of southwest China (Yunnan, Sichuan, Guizhou, Chongqing, and Xizang) between 2000 and 2024 (21°08′–33°41′ N, 97°21′–110°11′ E). Only survey sites where both fleas and sucking lice were investigated were included, while sites with only one insect group or ectoparasitic mites were excluded.

Simultaneous investigations of fleas and sucking lice were conducted at 10 survey sites in eastern Xizang Autonomous Region. Most areas of Xizang were not covered due to high altitude, harsh climatic conditions, limited accessibility, and logistical constraints. Field surveys were conducted mainly from March to May and from September to November to avoid the rainy season and extreme winter conditions.

### Rodent trapping and ectoparasite collection

At each survey site, cage traps (18 × 12 × 9 cm; Guixi Mousetrap Apparatus Factory, Jiangxi, China) were placed in indoor habitats (human dwellings, barns, and stables) and outdoor habitats (farmlands, shrublands, and woodlands). Trap bait was adjusted by habitat type, with corn or peanuts used outdoors and steamed bread or fried dough used indoors.

In open habitats, traps were arranged in straight lines, spaced 5 m apart within lines and 20 m between lines, with 25 traps per line. Indoors, traps were placed at approximately 15 m² intervals along walls. An average of 200 traps were set daily for 15 consecutive days at each site, resulting in approximately 3,000 trap placements per site to ensure methodological consistency and comparability [38, 41].

Captured rodents were collected the following morning, individually placed in cloth bags, and transported to the laboratory. Fleas and sucking lice were collected by combing and manual inspection of each rodent over a white tray. Ectoparasites from each host were preserved separately in 70%–75% ethanol. Rodents were identified morphologically using standard external measurements, and host sex and age were determined based on body size, pelage characteristics, genital development, and anogenital distance [42–44]. All instruments were disinfected with 75% ethanol (Wuhan Jisi Instruments & Equipment, China) between examinations, and field personnel used appropriate personal protective equipment [2].

### Preparation and taxonomic identification of insects

Collected fleas and sucking lice were digested in 5% or 10% sodium hydroxide (Shanghai Macklin Biochemical Technology, China) or potassium hydroxide (Shanghai Aladdin Biochemical Technology, China), dehydrated through a graded ethanol series (30%–100%), clarified in ethanol–xylene (Shanghai Aladdin Biochemical Technology) (1:1, v/v), then in xylene, and mounted on glass slides (Jiangsu Qipinsheng Medical Supplies Co., Ltd, China) with Canadian balsam (Shanghai Macklin Biochemical Technology) or equivalent mounting media [16, 45]. After oven drying, specimens were examined under an Olympus CX31 trinocular microscope (Olympus Corporation, Tokyo, Japan). Species identification was performed using standard taxonomic keys and the literature, and all identifications were independently verified by multiple taxonomists [16, 46, 47]. Damaged or unidentifiable specimens were excluded from statistical analyses.

### Infestation indices and statistical analysis

Infestation was assessed using the constituent ratio (*C_r_*), prevalence (*P_M_*), mean abundance (*MA*), and mean intensity (*MI*). *P_M_* is the percentage of hosts infested with ectoparasitic insects. *MA* is the average number of insects per examined host, and *MI* is the average number of insects per infested host.

The indices were calculated as follows [38, 39]:

*C_r_* = (*N_i_* / *N*) × 100%

*P_M_* = (*H_i_* / *H*) × 100%

*MA* = *N_i_* / *H*

*MI* = *N_i_* / *H_i_*

where *N_i_* is the number of individuals of insect species *i*, *N* is the total number of insects collected, *H* is the total number of hosts examined, and *H_i_* is the number of infested hosts.

Differences in *P_M_* were analyzed using the chi-square (*χ*²) test. *MA* and *MI* were compared using nonparametric tests (Mann–Whitney U test or Kruskal–Wallis H test), with Bonferroni correction for multiple comparisons. Statistical significance was set at p < 0.05. All analyses were performed using SPSS 26.0 software (IBM Corporation, NY, USA).

### Community structure analysis

Community characteristics were evaluated using species richness (*S*), Shannon–Wiener diversity index (*H’*), Pielou evenness index (*E*), Simpson dominance index (*D*), and Berg–Parker dominance index (*d*), as follows [48]:

*H’* = −Σ (*N_i_* / *N*) ln (*N_i_* / *N*)

*E* = *H’* / ln *S*

*D* = Σ (*N_i_* / *N*)²

*d* = *N*_max_ / *N*

where *N_i_* is the number of individuals of species *i*, *N* is the total number of individuals, *N*_max_ is the number of individuals of the most abundant species, and ln denotes the natural logarithm.

### Association between fleas and sucking lice

The association coefficient (*V*) was used to evaluate the relationship between flea and sucking louse infestations in *R. tanezumi* [49]:

*V* = (*ad* − *bc*) / √[(*a* + *b*)(*a* + *c*)(*b* + *d*)(*c* + *d*)]

where *a* represents hosts infested with both fleas and lice, *b* represents hosts infested with lice only, *c* represents hosts infested with fleas only, and *d* represents hosts free of both groups. Values of *V* range from −1 to +1. Statistical significance was assessed using the chi-square test.

### Interspecific relationship analysis

Spearman’s rank correlation coefficient (*r*) was used to assess interspecific relationships among flea and louse species [50]:

*r* = Σ (*X_i_* − *X̅*)(*Y_i_* − *Y̅*) / √[Σ (*X_i_* − *X̅*)² Σ (*Y_i_* − *Y̅*)^2^]

where *X_i_* and *Y_i_* are observed values of variables *X* and *Y*, *X̅* and *Y̅* are their respective means, and *n* is the number of samples. Heat maps were generated using Origin 2024 software (OriginLab Corporation, USA) to visualize correlation patterns.

### Host relative fatness assessment

The nutritional status of *R. tanezumi* was evaluated using the relative fatness index (*K*), calculated as [39, 51–53]:

*K* = 100*W* / *L*³

where *W* is body weight (g) and *L* is body length (cm). Individual *K* values were calculated for each rat, and the mean *K* value (mean ± standard deviation) was used as a threshold to classify hosts into low-fatness and high-fatness groups for comparative analyses.

## RESULTS

### Overall infestation and distribution of ectoparasitic insects

**Figure 1:**
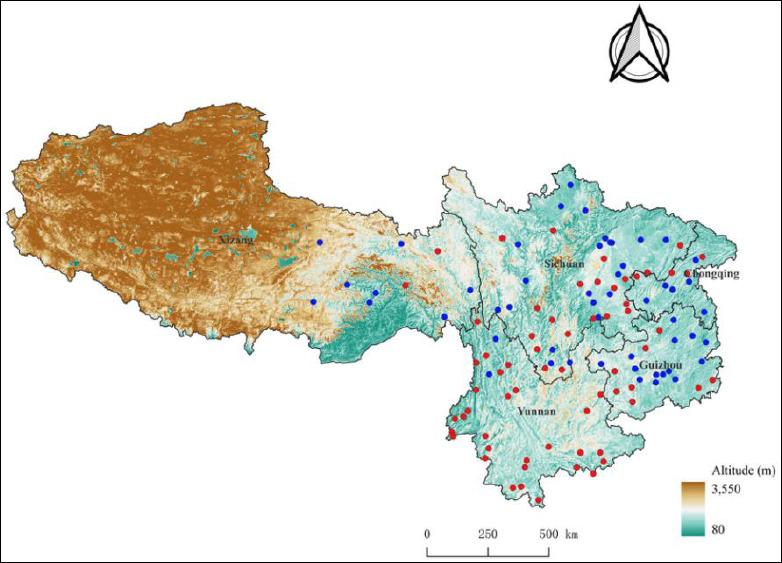
Field survey sites for ectoparasitic insects (fleas and sucking lice) on *Rattus tanezumi* rats across the five provincial regions of southwest China (2000-2024). The “
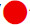
” represents the sites where *R. tanezumi* rats were found (n = 64), and “
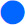
” stands for the sites without *R. tanezumi* found (n = 52). The total number of survey sites was 116 (64 + 52 = 116). The map was created using QGIS 3.40.4 software with the WGS84 coordinate system (EPSG code: 3857), and the administrative boundaries were obtained from the National Geospatial Information Service Platform of China.

A total of 3,069 *R. tanezumi* were captured from 64 of the 116 survey sites (Figure 1). Among these 64 sites, ectoparasitic insect infestation was detected at 51 sites, including 32 sites where rats were co-infested with two insect groups (fleas and sucking lice), 12 sites with flea-only infestations, and 7 sites with louse-only infestations, for a total of 51 infested sites (32 + 12 + 7 = 51). Of the 3,069 *R. tanezumi* examined, 1,240 were infested with ectoparasitic insects, and a total of 12,539 insect specimens were collected. The 1,240 infested rats comprised 131 individuals co-infested with fleas and lice, 486 infested only with fleas, and 623 infested only with lice (131 + 486 + 623 = 1,240). All ectoparasitic insects were taxonomically identified as belonging to 7 families, 25 genera, and 34 species within two orders, Siphonaptera (fleas) and Phthiraptera (sucking lice). The overall infestation indices for ectoparasitic insects (fleas + lice) on *R. tanezumi* were *P_M_* = 40.40%, *MA* = 4.09 insects per examined host, and *MI* = 10.11 insects per infested host. Among the 34 insect species identified, 10 flea species are known or potential vectors of zoonotic diseases, including plague, murine typhus, flea-borne spotted fever, and bartonellosis. These vector flea species were *Xenopsylla cheopis*, *Leptopsylla segnis*, *Monopsyllus anisus*, *Pulex irritans*, *Leptopsylla serinus*, *Stenoponia apertus*, *Ctenocephalides felis*, *Paradoxopsyllus custodis*, *Frontopsylla spadix*, and *Neopsylla specialis*. Among them, *X. cheopis* is the most important vector of plague and murine typhus in southwest China. Details of these vector flea species and the zoonotic diseases they transmit are presented in Table 1. [8, 10–12, 14, 16, 54–62].

**Table 1:** The ten vector flea species on *Rattus tanezumi* in southwest China (2000-2024) and their transmission of zoonotic diseases.

Names of the vector flea species	Number of fleas and constituent ratios (*C_r_*, %)		Associated zoonotic diseases transmitted by fleas	References

	No.	*C_r_* (%)		
*Xenopsylla cheopis* (Rothschild, 1903)	1599	69.49	Plague, murine typhus, flea-borne spotted fever, bartonellosis, cestodiasis	[8, 10, 12, 14, 16, 54, 55]
*Leptopsylla segnis* (Schönherr, 1811)	483	20.99	Plague, murine typhus, flea-borne spotted fever, bartonellosis, cestodiasis	[8, 14, 16, 54–56]
*Ctenocephalides felis* (Bouche, 1835)	3	0.13	Plague, murine typhus, flea-borne spotted fever, bartonellosis, cestodiasis, feline leukemia, allergic dermatitis, helminthiasis	[8, 10–12, 14, 16, 55, 57]
*Monopsyllus anisus* (Rothschild, 1907)	31	1.35	Plague, pseudotuberculosis, listeriosis, swine erysipelas	[16, 54, 58]
*Pulex irritans* Linnaeus, 1758	2	0.09	Plague, murine typhus, flea-borne spotted fever, cestodiasis	[8, 10, 12, 14, 16, 54, 57]
*Neopsylla specialis* (Jordan, 1932)	16	0.70	Plague	[8, 16, 59, 60]
*Frontopsylla spadix* (Jordan *et* Rothschild, 1921)	48	2.09	Plague	[16, 59–61]
*Stivalius aporus* Jordan *et* Rothschild, 1922	25	1.09	Plague	[16, 62]
*Lentistivalius serinus* (Rothschild, 1908)	5	0.22	Plague	[16]
*Paradoxopsyllus custodis* (Jordan, 1932)	89	3.87	Plague	[16]
Total	2301	100.00		

### Comparison of flea and sucking louse infestation

Of the 12,539 ectoparasitic insects collected from *R. tanezumi*, 2,442 fleas were identified as 30 species, 23 genera, and five families within the Order Siphonaptera, whereas 10,097 sucking lice were identified as four species, two genera, and two families within the Order Phthiraptera. Flea species accounted for 88.24% of all insect species but only 19.48% of individuals, whereas sucking lice represented 11.76% of species and 80.52% of individuals. Compared with sucking lice (four species with 10,097 individuals), fleas exhibited markedly higher species richness (30 spp.) but lower individual abundance (2,442 individuals). The overall infestation indices for fleas were *P_M_* = 20.10%, *MA* = 0.80, and *MI* = 3.96, whereas those for sucking lice were *P_M_* = 24.57%, *MA* = 3.29, and *MI* = 13.39. The infestation indices for sucking lice were significantly higher than those for fleas (p < 0.05).

### Taxonomic composition and dominant flea species

**Figure 2:**
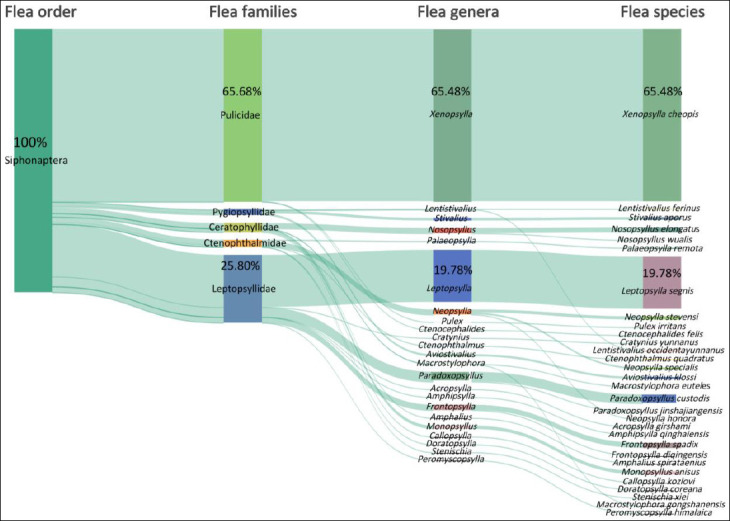
Visualization of constituent ratios (C_r_) of 2,442 fleas at different taxonomic levels (orders, families, genera, and species) on *Rattus tanezumi* rats (hosts) in southwest China (2000-2024). The shade width of each patch (band or string) represents the corresponding constituent ratio (C_r_) of fleas at a certain taxonomic level, order, family, genus, or species.

A total of 617 *R. tanezumi* rats were infested with fleas, including 486 rats infested only with fleas and 131 rats co-infested with fleas and sucking lice. The hierarchical taxonomic structure of fleas and the proportional distribution of flea individuals across taxonomic levels were visualized using a Sankey diagram (Figure 2), illustrating the constituent ratios of 2,442 flea specimens across one order, five families, 23 genera, and 30 species. At the family and genus levels, Pulicidae (*C_r_* = 65.68%) and *Xenopsylla* (*C_r_* = 65.48%) were dominant, followed by Leptopsyllidae (*C_r_* = 25.80%) and *Leptopsylla* (*C_r_* = 19.78%). At the species level, *X. cheopis* and *L. segnis* were the dominant flea species, jointly accounting for 85.26% of total flea individuals (Table 2, Figure 2). Among the 30 flea species identified, the infestation indices of *X. cheopis* (*P_M_* = 13.16%, *MA* = 0.52, *MI* = 3.96) were significantly higher than those of other flea species (p < 0.05) (Table 2).

**Table 2:** Infestation indices of the main flea and sucking louse species on *Rattus tanezumi* in southwest China (2000-2024).

Main flea and sucking louse species	Number of hosts		No. and *C_r_* of fleas and sucking lice			Infestation indices of fleas and sucking lice	
		
	Examined	Infested	No.	*C_r_* (%)	*P_M_* (%)	*MA*	*MI*
*Xenopsylla cheopis*	3069	404	1599	65.48	13.16**	0.52**	3.96*
*Leptopsylla segnis*	3069	143	483	19.78	4.66**	0.16**	3.38*
Total		547	2082	100.00	17.82	0.68	3.81
*Hoplopleura pacifica*	3069	512	6564	64.98	16.68*	2.14*	12.82*
*Polyplax spinulosa*	3069	440	3496	34.61	14.34*	1.14*	7.95*
Total		952	10060	100.00	31.02	3.28	10.57

C_r_= constitutive ratio (%), *P*_M_=prevalence (%), *MA*=mean abundance (insects/per examined rat host), and *MI*=mean intensity (insects/per infested rat host). The asterisks of "*" and "**" represent p < 0.05 and p < 0.001, respectively.

### Taxonomic composition and dominant sucking louse species

**Figure 3:**
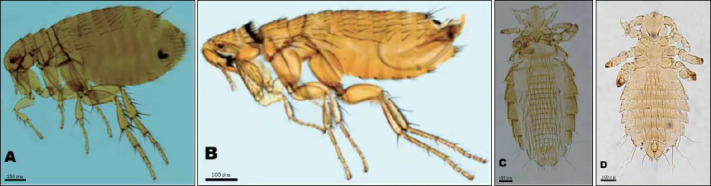
Photographs of four dominant insect species identified from *Rattus tanezumi* rats (hosts) in southwest China (2000-2024). (A) Xenopsylla cheopis (Rothschild, 1903) (♀, 10×20). (B) *Leptopsylla segnis* (Schönherr, 1811) (♂, 10×20). (C) *Hoplopleura pacifica* (Ewing, 1924) (♀, 10×20). (D) *Polyplax spinulosa* (Burmeister, 1839) (♂, 10×20).

A total of 754 *R. tanezumi* rats were infested with sucking lice, including 623 rats infested only with lice and 131 rats co-infested with lice and fleas. Among the two louse families and genera identified, Hoplopleuridae and *Hoplopleura* accounted for 65.01% of the total lice, whereas Polyplacidae and *Polyplax* accounted for 34.99%. Of the four louse species recorded, *Hoplopleura pacifica* was dominant, representing 65.01% (6,564/10,097) of total lice, followed by *Polyplax spinulosa* at 34.62% (3,496/10,097). The infestation indices of *H. pacifica* (*P_M_* = 16.68%, *MA* = 2.14, *MI* = 12.82) were significantly higher than those of *P. spinulosa* (*P_M_* = 14.34%, *MA* = 1.14, *MI* = 7.95) (p < 0.05). Moreover, the infestation indices (*P_M_*, *MA*, and *MI*) of *H. pacifica* were significantly higher than those of the two dominant flea species (p < 0.05) (Table 2). Representative micrographs of two dominant flea species (*X. cheopis* and *L. segnis*) and two dominant louse species (*H. pacifica* and *P. spinulosa*) are shown in Figure 3.

### Host sex-, age-, and nutrition-related infestation patterns

**Figure 4:**
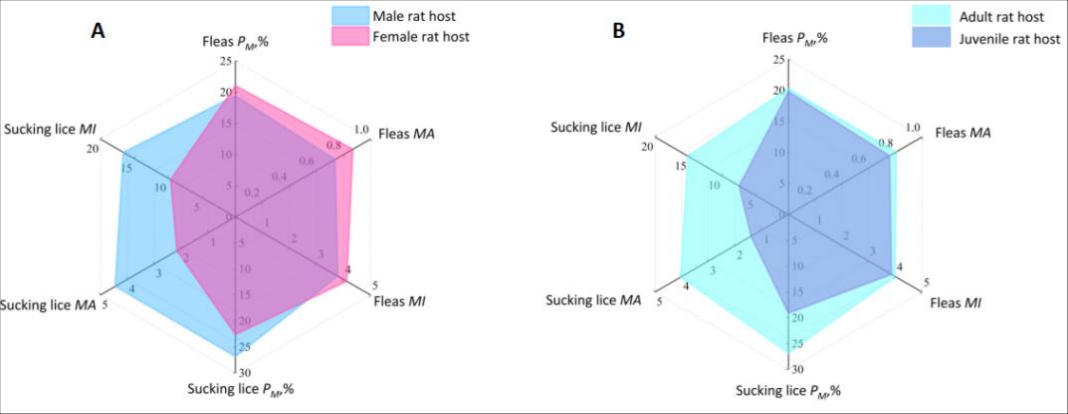
Radar chart visualization for insect infestations on different sexes and ages of rat hosts (*Rattus tanezumi*) in southwest China (2000-2024). (A) Insect infestations on different sexes of rat hosts (*R. tanezumi*): the blue area represents the infestation indexes (*P_M_, MA, and MI*) of insects (fleas and sucking lice) on male rat hosts and the pink area represents the P_M_, MA, and MI of insects on female rat hosts. (B) Insect infestations on different ages of rat hosts (*R. tanezumi*): the light blue area represents the infestation indexes (*P_M_, MA, and MI*) of insects on adult rat hosts, and the dark blue area represents the P_M_, MA, and MI of insects on juvenile rat hosts.

Of the 3,069 *R. tanezumi* rats examined, 3,042 had sex records (1,517 males and 1,525 females), and 3,061 had age records (2,197 adults and 864 juveniles). Infestation differences by sex and age were visualized using radar charts (Figure 4). Male rats exhibited higher constituent ratio, prevalence, and *MA* of sucking lice (*C_r_* = 67.11%, *P_M_* = 26.83%, *MA* = 4.45) than female rats (*C_r_* = 32.89%, *P_M_* = 22.62%, *MA* = 2.17) (p < 0.05). Adult hosts showed higher *C_r_* (87.92%) and infestation indices (*P_M_* = 26.81%, *MA* = 4.04, *MI* = 15.07) than juvenile hosts (*P_M_* = 19.10%, *MA* = 1.41, *MI* = 7.39) (p < 0.001). In contrast, differences in flea infestation indices between sexes and age groups were not statistically significant (p > 0.05) (Figure 4).

Based on relative fatness (*K*), rats were categorized into low-fatness (*K* = 2.29 ± 0.33 g/cm³) and high-fatness (*K* = 3.52 ± 0.87 g/cm³) groups. Flea prevalence was higher in the low-fatness group (*P_M_* = 17.88%) than in the high-fatness group (*P_M_* = 13.02%), whereas *MA* was lower (*MA* = 0.51 vs. 0.57) (p < 0.05). For sucking lice, both *P_M_* and *MA* were significantly higher in the low-fatness group (*P_M_* = 27.87%, *MA* = 5.16) than in the high-fatness group (*P_M_* = 18.93%, *MA* = 2.31) (p < 0.001). *MI* did not differ significantly between nutritional groups for either insect group (p > 0.05) (Table 3).

**Table 3:** Infestation indices of fleas and sucking lice on *Rattus tanezumi* with different nutritional statuses in southwest China (2000-2024).

Ectoparasitic insects	Different nutritional statuses of *R. tanezumi* hosts	Number of examined hosts	No. of infested hosts	No. and *C_r_* of ectoparasitic insects		Infestation indices of ectoparasitic insects on hosts of *R. tanezumi*		
	
				No.	*C_r_* (%)	*P_M_* (%)	*MA*	*MI*
Fleas	Low-fatness group	1141	204	586	58.02	17.88*	0.51*	2.87
	High-fatness group	745	97	424	41.98	13.02*	0.57*	4.37
	Total	1886	301	1010	100.00	15.96	0.54	3.36
Sucking lice	Low-fatness group	1141	318	5891	77.37	27.87**	5.16 **	18.53
	High-fatness group	745	141	1723	22.63	18.93**	2.31 **	12.22
	Total	1886	459	7614	100.00	24.34	4.04	16.59

The *C_r_, P_M_, MA, MI*, ”*” and ”**” are the same as in Table 2.

### Environmental variation in infestation patterns

**Figure 5:**
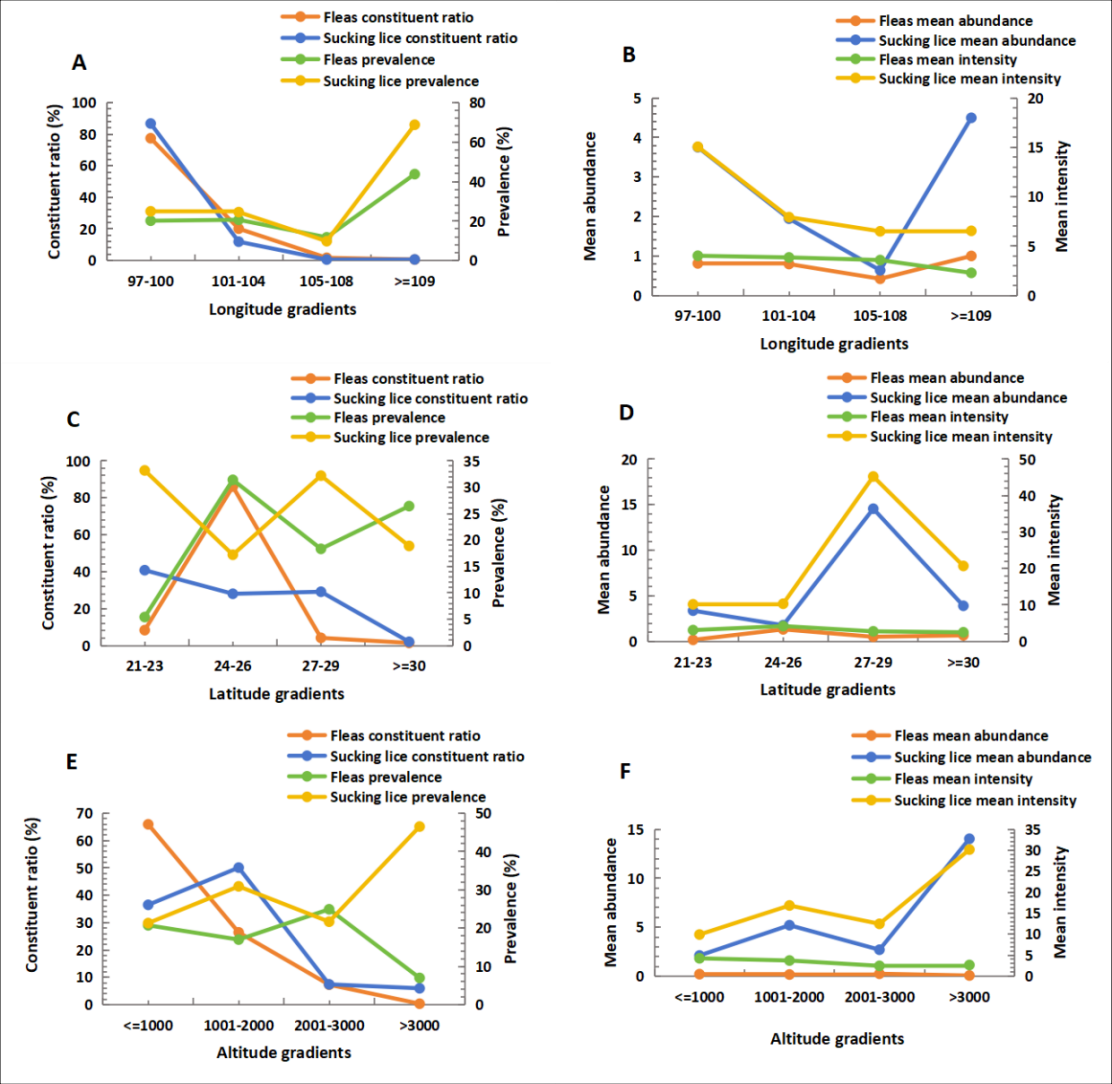
Fluctuations of insects (fleas and sucking lice) infesting *Rattus tanezumi* along different gradients of longitude (A and B), latitude (C and D), and altitude (E and F) in southwest China (2000-2024).

Infestation indices of fleas and sucking lice varied across environmental gradients (Figure 5). Along longitude gradients, both insect groups had the highest *C_r_* at 97–100°E, whereas the highest *P_M_* and *MA* occurred at ≥109°E (p < 0.05). Along latitude gradients, fleas showed the highest *C_r_*, *P_M_*, and *MA* at 24–26°N (p < 0.001). Sucking lice exhibited the highest *C_r_* and *P_M_* at 21–23°N (p < 0.001) and the highest *MA* at 27–29°N (p < 0.001). Along altitude gradients, fleas showed the highest *C_r_* and *MI* at ≤1000 m (p < 0.05) but the highest *P_M_* and *MA* at 2001–3000 m (p < 0.05). Sucking lice had the highest *P_M_*, *MA*, and *MI* at >3000 m (p < 0.05) (Figure 5). Habitat-based analyses indicated that fleas had significantly higher *P_M_* and *MA* in indoor habitats, whereas sucking lice showed higher *C_r_*, *P_M_*, and *MA* in outdoor habitats (p < 0.001). Fleas exhibited higher *C_r_*, *P_M_*, and *MA* in flatland landscapes (p < 0.001), whereas sucking lice showed higher infestation indices in mountainous landscapes (p < 0.05) (Table 4).

**Table 4:** Infestation indices of fleas and sucking lice on *Rattus tanezumi* in southwest China (2000-2024).

Ectoparasitic insects	Different habitats and landscapes	Number of examined hosts	No. of infested hosts	No. and *C_r_* of ectoparasitic insects		Infestation indices of ectoparasitic insects on the *R. tanezumi* host		
	
				No.	*C_r_* (%)	*P_M_* (%)	*MA*	*MI*
Fleas	**Habitats**							
	Indoor	637	212	841	34.44	33.28**	1.32**	3.97
	Outdoor	2432	405	1601	65.56	16.65**	0.66**	3.95
	Total	3069	617	2442	100.00	20.10	0.80	3.96
	**Landscapes**							
	Mountainous	1230	196	588	26.09	15.93**	0.48**	3.00*
	Flatland	1780	396	1666	73.91	22.25**	0.94**	4.21*
Sucking lice	**Habitats**							
	Indoor	637	58	737	7.30	9.11**	1.16**	12.71
	Outdoor	2432	696	9360	92.70	28.62**	3.85 **	13.45
	Total	3069	754	10097	100.00	24.57	3.29	13.39
	**Landscapes**							
	Mountainous	1230	340	6649	65.96	27.64*	5.41 *	19.56
	Flatland	1780	406	3431	34.04	22.81*	1.93*	8.45
	Total	3010	746	10080	100.00	24.78	3.35	13.51

The *C_r_, P_M_, MA, MI*, ”*” and ”**” are the same as in Table 2.

### Comparison of flea and sucking louse community indices

The flea community on *R. tanezumi* was characterized by high species richness (30 species) but low individual abundance (2,442 individuals), whereas the louse community comprised only four species with high abundance (10,097 individuals). Consequently, flea communities showed higher species richness and Shannon–Wiener diversity index, whereas louse communities exhibited higher Pielou evenness and Simpson dominance indices (Table 5).

**Table 5:** Community indices of fleas and sucking lice on *Rattus tanezumi* in southwest China (2000-2024).

Ectoparasitic insects	Number of insects	Community indexes				

		*S*	*H’*	*E*	*D*	*d*
Fleas	2442	30	1.24	0.37	0.47	0.65
Sucking lice	10097	4	0.67	0.48	0.54	0.65

S = Richness, *H*' = Shannon-Wiener diversity index, *E* = Pielou evenness index, *D* = *Simpson* dominance index, and *d* = Berger-Parker index.

### Sex and age structure of ectoparasitic insects

**Figure 6:**
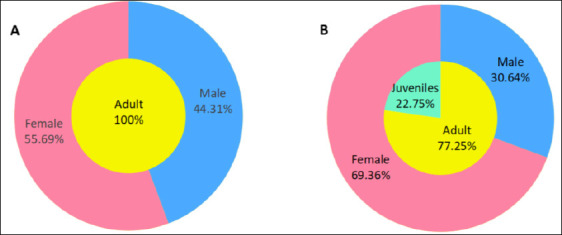
Sex and age structure of ectoparasitic insects (fleas and sucking lice) on *Rattus tanezumi* in southwest China (2000-2024). (A) Sex and age structure of fleas. (B) Sex and age structure of sucking lice.

Within flea and louse populations, females had higher constituent ratios than males. Adult lice accounted for a substantially higher proportion of the louse population than juvenile lice (Figure 6). In contrast, only adult fleas were observed because juvenile flea stages are not ectoparasitic.

### Mutual relationship between flea and sucking louse infestations

The association coefficient (*V*) was used to assess the relationship between flea and sucking louse infestations, based on the contingency data presented in Table 6. The calculated value of *V* was −0.0389, close to zero and statistically significant (p < 0.05), indicating an essentially independent distribution of fleas and sucking lice on *R. tanezumi* (Table 6).

**Table 6:** Contingency table for analyzing the mutual relationship between fleas and sucking lice on *Rattus tanezumi* in southwest China (2000-2024).

Frequency of fleas and sucking lice on *Rattus tanezumi*		Fleas (*X*)		Total

		+	-	
Sucking lice (*Y*)	+	131 (*a*)	623 (*b*)	754 (*a* + *b*)
	-	486 (*c*)	1829 (*d*)	2315 (*c* + *d*)
	Total	617 (*a* + *c*)	2452 (*b* + *d*)	3069 (*n*)
*V* and the chi-square test		*V*=−0.0389, *χ*^2^=4.64, p < 0.05		

### Interspecific relationships among dominant insect species

**Figure 7:**
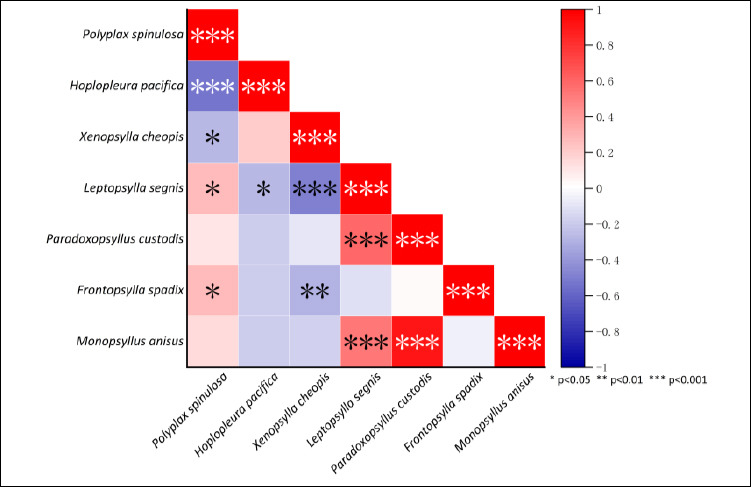
Heat map visualization for the relationships among the main flea and sucking louse species on *Rattus tanezumi* in southwest China (2000-2024).

Interspecific relationships among the seven dominant insect species (five flea species and two louse species) were assessed using Spearman’s rank correlation coefficient and visualized as a heatmap (Figure 7). Positive correlations (0 < *r* < 1) are shown in red, and negative correlations (−1 < *r* < 0) are shown in blue, with significance levels indicated as ***p < 0.001, **p < 0.01, and *p < 0.05. The analysis revealed both positive and negative associations among species. For example, *M. anisus* and *P. custodis* showed a strong positive correlation (*r* = 0.90, p < 0.001), whereas *H. pacifica* and *P. spinulosa* exhibited a significant negative correlation (*r* = −0.53, p < 0.001) (Figure 7).

## DISCUSSION

### Epidemiological context of southwest China

Southwest China, as defined in this study, comprises five provincial regions (Yunnan, Guizhou, Sichuan, Chongqing, and Xizang) and covers a vast area of approximately 2,341,467 km², with a population of about 205 million. This region is a natural focus for numerous zoonotic diseases, including plague, murine typhus, bartonellosis, leptospirosis, HFRS, and scrub typhus. Historically, plague and murine typhus, two major flea-borne diseases, were prevalent in Yunnan, Sichuan, and Xizang, with successive human cases reported over extended periods [63–68]. More recent studies have identified Yunnan and Sichuan as important endemic areas for bartonellosis, with rodents, cats, and companion animals as the primary sources of infection [28, 31]. Notably, 1,157 human cases of murine typhus were reported in Xishuangbanna Prefecture, Yunnan Province, in 2011 alone, corresponding to an incidence of 102.10 per 100,000 population [66]. The oriental house rat (*R. tanezumi*) is not only a common agricultural pest but also a significant medical and veterinary pest because of its close association with a wide range of zoonotic diseases [32, 33]. The pathogens responsible for these diseases can be readily transmitted among rat populations and from rats to humans or domestic animals via the blood-feeding activity of ectoparasitic insects, particularly fleas [8, 10, 13]. Consequently, investigating the infestation and distribution of ectoparasitic insects on *R. tanezumi* in southwest China is of considerable medical and veterinary importance.

### Novelty and scope of the present study

Previous studies in southwest China have primarily focused on ectoparasitic mites, including chiggers and gamasid mites, on *R. tanezumi* [2, 3, 34, 40], while ectoparasitic insects have not been comprehensively analyzed due to limited taxonomic coverage. Earlier parasitological investigations in this region typically concentrated on mites or, in some cases, on fleas as a single insect group [54, 69]. In contrast, the present study is the first to simultaneously investigate fleas and sucking lice parasitizing *R. tanezumi* across five provinces in southwest China. The original data were derived from a multi-provincial field investigation conducted between 2000 and 2024 (Figure 1). The extensive spatial coverage, encompassing 116 survey sites, provides a large-scale ecological dataset on rodent-associated ectoparasites across this vast territory. In total, 3,069 *R. tanezumi* individuals were captured at 64 of the 116 survey sites, and these sites were distributed across all five provincial regions (Figure 1), further confirming that *R. tanezumi* is a widely distributed and dominant rodent species in southwest China [2, 3]. In this study, 34 species and 12,539 individuals of ectoparasitic insects (fleas and sucking lice) were identified from *R. tanezumi*, with overall infestation indices of *P_M_* = 40.40%, *MA* = 4.09 insects per examined host, and *MI* = 10.11 insects per infested host. These results clearly demonstrate that *R. tanezumi* is highly susceptible to infestation by both fleas and sucking lice.

### Vector flea diversity and zoonotic implications

Among the 34 ectoparasitic insect species identified, 30 were flea species and only four were louse species, indicating substantially higher species diversity in fleas than in sucking lice. Globally, nearly 3,000 flea species have been described, with more than 600 species recorded in China [70–72]. A considerable proportion of flea species are recognized vectors of zoonotic pathogens, including those causing plague, murine typhus, flea-borne spotted fever, and bartonellosis [7–16]. In the present study, 10 of the 30 flea species identified on *R. tanezumi* were confirmed as vector species capable of transmitting plague, murine typhus, and other zoonotic diseases [54–62]. These species included *X. cheopis, L. segnis, M. anisus, P. irritans, L. serinus, S. apertus, C. felis, P. custodis, F. spadix*, and *N. specialis* (Table 1). In southwest China, plague occurs in both domestic rodent and wild rodent foci. *X. cheopis* is the principal vector in domestic rodent plague foci and also the major vector of murine typhus in these areas and surrounding regions [16]. In contrast, *F. spadix* and *N. specialis* are the dominant vectors in wild rodent plague foci [60, 61]. Moreover, *X. cheopis, P. irritans, C. felis*, and *L. segnis* also function as intermediate hosts for *Hymenolepis nana*, *Hymenolepis diminuta*, and *Dipylidium caninum* [8, 10–12, 14, 16, 55–57]. To date, no previous study has reported such a comprehensive assemblage of ten vector flea species on a single rodent host in southwest China. This finding provides new evidence of multi-vector coexistence and highlights potential zoonotic transmission hotspots associated with *R. tanezumi* populations.

### Comparative infestation patterns of fleas and sucking lice

Compared with sucking lice (four species comprising 10,097 individuals), fleas exhibited high species richness (30 species) but low individual abundance (2,442 individuals) on *R. tanezumi*. Overall infestation indices for sucking lice (*P_M_* = 24.57%, *MA* = 3.29, *MI* = 13.39) were significantly higher than those for fleas (*P_M_* = 20.10%, *MA* = 0.80, *MI* = 3.96) (p < 0.05). Despite the heavier infestation burden of sucking lice, the flea community showed substantially higher species richness (*S*) and Shannon–Wiener diversity index (*H’*) than the louse community (Table 5). This contrast reflects fundamental differences in community structure, with the louse community characterized by low species diversity and high dominance. Such patterns are closely related to life-history traits and host specificity. Sucking lice are obligate ectoparasites of eutherian mammals that permanently reside on their hosts throughout all life stages, resulting in high host specificity developed through long-term coevolution [73–75]. In contrast, fleas possess a four-stage life cycle, with only adult stages parasitic on warm-blooded animals, and many flea species exhibit low host specificity [14, 76–82]. In the present study, as many as 30 flea species were found on *R. tanezumi*, whereas only four louse species were identified, further illustrating these ecological differences. The lower host specificity of fleas facilitates pathogen transmission among diverse wild and domestic hosts, including humans [83, 84], whereas the high host specificity of sucking lice limits their inter-host transmission potential [85, 86].

### Host sex-, age-, and nutrition-related infestation patterns

Significant sex- and age-related biases were observed in sucking louse infestation. Male *R. tanezumi* exhibited significantly higher constituent ratios and infestation indices (*P_M_* and *MA*) than females (p < 0.05), and adult rats showed higher *C_r_* and infestation indices (*P_M_*, *MA*, and *MI*) than juveniles (p < 0.001). In contrast, flea infestation indices did not differ significantly among host sexes or age groups (p > 0.05) (Figure 4). These patterns suggest that host physiological status has a stronger influence on obligate ectoparasites such as sucking lice than on fleas. Similar sex- and age-related biases in parasitic infections have been reported previously [87, 88] and may be attributed to differences in energy expenditure, immune function, body surface area, and exposure risk between males and females or between adults and juveniles [89, 90].

Host nutritional status also influenced ectoparasite infestation. Relative fatness (*K*), a widely used index of host nutritional condition [39, 51, 52], showed that rats with poor nutrition (low-fatness) had significantly higher *P_M_* and *MA* of sucking lice than those with better nutritional status (p < 0.001) (Table 3). This finding provides novel evidence that poor nutritional condition increases susceptibility to louse infestation in *R. tanezumi*. In contrast, the effect of host nutrition on flea infestation appeared inconsistent. Although flea *P_M_* was higher in the low-fatness group (p < 0.05), *MA* was lower in the high-fatness group (p < 0.05), suggesting that the relationship between flea infestation and host nutrition may be complex and warrants further investigation.

### Environmental heterogeneity of ectoparasite infestation

Beyond host-related factors, ectoparasite infestation was strongly influenced by environmental conditions. Infestation indices for fleas and sucking lice varied along gradients of longitude, latitude, altitude, habitat type, and geographical landscape (Table 4, Figure 5), reflecting pronounced environmental heterogeneity within the same host species. These findings are consistent with previous studies showing that ectoparasite infestation can vary substantially under different environmental conditions, even within the same host species [41,91]. Integrating multiple environmental gradients in the present study identifies niche preferences and habitat-dependent infestation patterns of fleas and sucking lice on *R. tanezumi* that have not been previously reported at this spatial scale.

### Interactions between flea and louse communities

The association coefficient (*V*) was used to assess the relationship between flea and sucking louse infestations in *R. tanezumi*. The calculated *V* value was close to zero (*V* = −0.0389, p < 0.05) (Table 6), indicating that infestations by fleas and sucking lice are essentially independent. This suggests that the presence of one insect group does not significantly influence infestation by the other, and that neither competitive exclusion nor facilitation occurs between these two ectoparasite groups on *R. tanezumi* [49, 92]. To our knowledge, this study is among the first to quantitatively demonstrate the independence of flea and louse distributions on a single rodent host using association coefficient analysis.

### Interspecific relationships and population structure

Heatmap-based interspecific correlation analysis using Spearman’s rank correlation coefficient revealed varying degrees of positive and negative associations among flea and louse species (Figure 7). Positive correlations indicate a tendency for co-occurrence on the same host, whereas negative correlations suggest potential interspecific competition during host selection [93, 94]. Within ectoparasite populations, female fleas and female sucking lice had higher constituent ratios than males (Figure 6), likely reflecting longer female lifespan in many insect species [95, 96]. The markedly higher proportion of adult lice relative to juveniles remains unexplained and highlights the need for further investigation into louse population dynamics.

### Study limitations and future perspectives

Although this retrospective study provides comprehensive insights into the infestation status, distribution patterns, and ecological characteristics of ectoparasitic insects on *R. tanezumi* in southwest China, several limitations should be acknowledged. Survey sites were not evenly distributed across all provincial regions, with only 10 sites in eastern Xizang, leaving large areas unsampled (Figure 1). The long study period (2000–2024) also introduces potential temporal variation related to climate and vegetation changes, which were not analyzed due to a lack of long-term environmental data. Additionally, this study focused on overall infestation patterns rather than seasonal dynamics. Detailed investigations of seasonal variation would require fixed-site, monthly sampling over at least one full year, representing a fundamentally different study design.

## CONCLUSION

This extensive, long-term research offers the first thorough evaluation of ectoparasitic insects affecting the oriental house rat (*R. tanezumi*) across five provinces in southwest China. Over 24 years, data from 116 survey sites uncovered 12,539 ectoparasites across 34 species, comprising 30 flea species and four sucking louse species. The infestation levels were notably high (*P_M_* = 40.40%, *MA* = 4.09, *MI* = 10.11), indicating that *R. tanezumi* is highly prone to ectoparasitic infestations. Importantly, ten flea species identified are either confirmed or potential vectors of key zoonotic diseases such as plague and murine typhus. Fleas demonstrated high species diversity but low abundance, while sucking lice showed the opposite, low diversity but high infestation severity. Variations related to host factors (sex, age, nutritional status) and environmental factors (longitude, latitude, altitude, habitat, landscape) were evident. Flea and louse infestations appeared largely independent, with interspecific analysis revealing both positive and negative co-occurrence patterns among dominant species.

The presence of multiple vector flea species on *R. tanezumi* emphasizes this rodent’s crucial role as a reservoir and amplifier of zoonotic diseases in southwest China. These results highlight the need for integrated surveillance of rodents and ectoparasites under a One Health approach, especially in areas with histories of plague and murine typhus. Controlling *R. tanezumi* populations and managing ectoparasites in homes and farms could significantly lower the risk of flea- and louse-borne diseases in humans and domestic animals.

This study’s key strengths include its extensive temporal range from 2000 to 2024, broad geographic coverage across five provincial regions, large sample size, and the use of standardized infestation indices. Analyzing both fleas and sucking lice simultaneously, along with host physiological factors and various environmental gradients, offers a comprehensive ecological perspective that was missing in earlier research. Additionally, identifying ten vector flea species on a single rodent host is a novel and epidemiologically significant contribution.

Several limitations must be recognized. Survey sites were unevenly spread throughout the study area, especially in Xizang, potentially affecting the spatial representativeness. The retrospective dataset limited the inclusion of detailed yearly climate and vegetation data, and the seasonal changes of ectoparasitic insects were not examined. Furthermore, molecular screening of pathogens in fleas and lice was outside the scope of this study.

Future research should include consistent, year-round sampling at fixed locations to better understand seasonal changes in flea and louse populations. Using detailed environmental data alongside molecular methods to detect zoonotic pathogens in ectoparasites can enhance risk assessments. Additionally, conducting comparative studies with more rodent hosts could help clarify host specificity, the potential for cross-species transmission, and how ectoparasite communities develop.

In summary, this research shows that *R. tanezumi* harbors a diverse and epidemiologically important group of ectoparasitic insects in southwest China. The differing ecological patterns between fleas and sucking lice, along with their links to host characteristics and environmental variability, offer valuable insights into rodent–ectoparasite relationships. These results provide a strong scientific basis for enhancing surveillance, prevention, and control efforts against rodent-borne and ectoparasite-related zoonotic diseases in the area.

## DATA AVAILABILITY

The data used in the study are available from the corresponding author on reasonable request.

## AUTHORS’ CONTRIBUTIONS

XJZ and YNL: Equal contributors to the study, software, formal analysis, visualization, methodology, data curation, and writing-original draft. XGG: conceptualization, validation, supervision, writing-review & editing, data curation, investigation, and specimen identification; TGR: methodology, investigation, and specimen making and identification. YGJ: methodology and investigation. LZ: methodology. TJQ: investigation. All authors have read and agreed to the published version of the manuscript.
